# Effect of Carvacrol on Diabetes-Induced Oxidative Stress, Fibrosis and Apoptosis in Testicular Tissues of Adult Rats

**DOI:** 10.33549/physiolres.935573

**Published:** 2025-06-01

**Authors:** Burcu GÜLTEKİN, Seda ÇETİNKAYA KARABEKİR, Ilknur ÇINAR AYAN, Hasan Basri SAVAŞ, Gökhan CÜCE, Sabiha Serpil KALKAN

**Affiliations:** 1 Department of Histology and Embryology, Faculty of Medicine, Necmettin Erbakan University, Konya, Republic of Türkiye; 2 Department of Histology and Embryology, Faculty of Medicine, Bakırçay University, Izmir, Republic of Türkiye; 3 Department of Medical Biology, Faculty of Medicine, Necmettin Erbakan University, Konya, Republic of Türkiye; 4 Department of Medical Biochemistry, Faculty of Medicine, Mardin Artuklu University, Mardin, Republic of Türkiye

**Keywords:** Diabetes Mellitus, Experimental, Testis, Carvacrol, Apoptosis, Fibrosis

## Abstract

Diabetes mellitus (DM) is a chronic and widespread disease that negatively affects the male reproductive system. Carvacrol (CAR), a naturally occurring flavonoid in plants, exhibits various biological and pharmacological activities, including anti-inflammatory, antioxidant, and anticancer properties. This study aimed to investigate the potential effects of CAR on testicular tissue damage induced by diabetes, which was modeled by Streptozotocin (STZ) administration. Thirty-two male Wistar albino rats were divided into four groups: Group 1: Control (n=8), Group 2: DM (n=8), Group 3: DM+DMSO (0.1 % dimethyl sulfoxide) (n=8), and Group 4: DM+CAR (20 mg/kg) (n=8). Diabetes was induced by a single intraperitoneal STZ injection (50 mg/kg). Histological changes were assessed using Hematoxylin-Eosin (H&E) staining and the Johnsen scoring system. Apoptosis was evaluated through immunohistochemical staining for the mitochondrial apoptosis markers Bax and Bcl-2, as well as RT-qPCR analysis of their gene expression levels. Fibrosis assessment involved Masson-Trichrome staining and RT-qPCR analysis of mRNA levels for the COL1A1 and COL3A1 genes. Additionally, Total Oxidant Status (TOS), Total Antioxidant Status (TAS), Oxidative Stress Index (OSI), and C-Reactive Protein (CRP) levels were measured in testicular tissue. CAR treatment significantly improved histological alterations associated with diabetes-induced testicular damage. DM was found to increase Bax levels while reducing Bcl-2 levels, whereas CAR reduced Bax levels and increased Bcl-2 gene and protein expression. TOS and OSI levels were elevated in the DM group, whereas TAS levels increased in the DM+CAR group. No significant differences in CRP levels were observed between the groups. These findings suggest that CAR may be effective in mitigating diabetes-induced testicular damage.

## Introduction

Diabetes mellitus (DM) is a metabolic disease that poses a serious threat to human health, with a high prevalence and decreasing age of onset globally. According to recent data from the International Diabetes Federation (IDF), the number of adults with diabetes has reached 463 million worldwide, a figure projected to rise to 578 million by 2030 [1].

One of the most commonly reported complications of diabetes in men is sexual dysfunction. DM causes detrimental changes in the reproductive system, reducing fertility rates in humans and animals through at least two mechanisms: oxidative stress and endocrine disruption. It has been shown that diabetes can negatively affect male reproductive parameters such as spermatogenesis, testicular tissue structure, sperm quality, testosterone (T) levels, and gonadotropin secretion rates. The adverse effects of diabetes on testicular function are thought to be related to insulin deficiency [2]. Patients with type 1 diabetes mellitus (DM1) exhibit reduced ejaculate volume due to the lack of normal epididymal contractions associated with mitochondrial damage, which subsequently decreases sperm motility. Attention is particularly directed toward DM1 as it commonly affects reproductive-age adults [3].

Moreover, studies have demonstrated that oxidative stress development in diabetic patients impairs testicular function. The primary functions of the testes are the production of testosterone and sperm, which are essential for maintaining the characteristics of males and species preservation. Reactive oxygen species (ROS) are known to impair testicular function [4]. Multiple studies on male diabetic animal models have reported that increased ROS levels lead to issues such as disruptions in Sertoli cell glucose metabolism, spermatogenesis, degenerative and apoptotic changes in the testes, dysregulation of testosterone synthesis and secretion, ejaculation dysfunction, and decreased libido [5]. Although insulin and oral antidiabetics are used in modern medicine to treat diabetes, challenges such as drug access, storage, administration, and side effects have driven a search for alternative, natural, or synthetic antidiabetic agents. In developing countries, traditional plant therapies are commonly used for diabetes management, and scientific research is increasingly focused on the hypoglycemic effects of medicinal plants [6]. Diabetic testicular dysfunction is a common complication in men with diabetes, contributing to reproductive challenges [7]. With the rising incidence of diabetes, these issues are becoming more prevalent. Oxidative stress and inflammation are key contributors to diabetes-induced testicular dysfunction [8–10]. The most significant source of oxidative stress in biological systems is the electron transport system located in the inner mitochondrial membrane [11]. Therefore, in our study, we assessed the mitochondrial apoptosis markers Bax and Bcl-2. Investigating these mechanisms is crucial for developing therapeutic strategies to improve fertility.

Carvacrol (CAR, C_10_H_14_O, 2-methyl-5-isopropylphenol) is a predominant monoterpenoid phenolic compound found in the essential oils of certain species within the Lamiaceae family. CAR possesses a wide range of biological and pharmacological properties, including antidiabetic, antibacterial, antifungal, anticancer, antiapoptotic, anti-inflammatory, hepatoprotective, and antioxidant activities [3]. Due to its anti-inflammatory and antioxidant properties, CAR is considered a potential treatment for diabetes [12,13]. CAR has been shown to provide protective effects in type 1 diabetes, including benefits for cardiac cells and function, blood parameters, vascular morphology and function, neurological damage, and testicular damage [14]. Although the protective and preventive effects of carvacrol against various pathophysiological conditions are well known, its therapeutic effect on diabetes-induced testicular damage has not been clearly established.

This study aims to investigate the potential protective effects of CAR against DM-induced oxidative stress and subsequent testicular tissue damage using histological, immunohistochemical, RT-qPCR, and biochemical analysis.

## Materials and Methods

### Experimental design

The study used 32 male Wistar Albino rats, 4 months old, weighing 250–300 g, obtained from the KONUDAM Experimental Medicine Application and Research Center of Necmettin Erbakan University. Ethical approval was granted by the Local Ethics Committee on Animal Experiments of Necmettin Erbakan University, under the decision numbered 2022–031 on July 6, 2022. The study adhered to the principles outlined in the Guide for the Care and Use of Laboratory Animals to protect animal rights. The animals were provided with tap water and standard laboratory feed, given *ad libitum*. They were housed in an environment with a temperature of 24±1 °C, humidity of 45±5 %, and a 12-hour light/dark cycle.

The rats were divided into 4 groups, each containing 8 animals:

Group 1 (Control): Citrate buffer (Bio-Optice Lot;1613, Milan MI, Italy) administration.
Group 2 (DM): Type 1 diabetes induced by streptozotocin (STZ, Sigma-Aldrich: S0130-1G, Missouri, USA).
Group 3 (DM+DMSO 0.1 %): Type 1 diabetes induced by STZ and treated daily with DMSO (0.1 %, Sigma-Aldrich: Lot; SZBF3010V, Missouri, USA) intraperitoneally for 4 weeks.
Group 4 (DM+CAR 20 mg/kg): Type 1 diabetes induced by STZ and treated daily with CAR (20 mg/kg, Sigma-Aldrich: Lot; SHBL6147, Missouri, USA) dissolved in 0.1 % DMSO and administered intraperitoneally for 4 weeks.

CAR was prepared fresh before each application. The CAR dose was chosen based on a previous study showing 20 mg/kg to be more effective than 10 mg/kg [15]. Diabetes induction was achieved by intraperitoneal injection of STZ prepared at 50 mg/kg in 0.01 M citrate buffer (pH=4.5) [16]. Three days post-STZ injection, blood glucose levels were measured using blood samples taken from the tail vein. Rats with blood glucose levels above 270 mg/dl were classified as diabetic. Weekly blood glucose monitoring was conducted throughout the study [5,6].

### Anesthesia procedure and sample collection

At the end of the experiment, blood and testis tissue samples were collected under anesthesia induced by an injection of Ketamine HCl (50 mg/kg) and Xylazine HCl (10 mg/kg). For histological examination, the left testis was fixed in 10 % formaldehyde solution for 48 h, while the right testis was stored at −80 °C for Bax and Bcl-2 expression analysis. Serum obtained from centrifuged blood samples collected in gelled tubes was aliquoted into Eppendorf tubes for biochemical parameter analysis. Tissue samples for biochemical analysis were frozen in aluminum foil. When measurements were taken, the tissues were thawed to room temperature, homogenized, and sonicated for biochemical analysis, measuring oxidative stress, and antioxidant balance parameters.

### Histological analysis

Tissue processing was performed using a device (Leica TP1020, Nussloch, Germany), with specific durations recorded for testes. Paraffin embedding was performed using a Leica HistoCore Arcadia H tissue embedding device (Nussloch, Germany), and 5 μm sections were obtained. Paraffin embedding was performed using a Leica HistoCore Arcadia H tissue embedding device (Nussloch, Germany), and 5 μm sections were obtained. For light microscopy evaluation, 10 different seminiferous tubules were selected for each preparation.

#### Hematoxylin & Eosin (H&E) staining

The sections were stained with Hematoxylin and Eosin (H&E) following routine procedures. To assess seminiferous tubule maturation and quality, modified Johnsen scoring was applied to slides evaluated at 400× magnification (Axiocam Erc 5s, Carl Zeiss AG, Germany). The ten-point scoring system is defined below (Table 1) [17].

#### Masson-Trichrome staining

Sections of 5 μm thickness were obtained from the testis blocks of experimental groups to assess fibrosis. Deparaffinization was conducted by passing the sections through xylene and a descending alcohol series (90 %, 80 %, 70 %, and 50 %). Masson’s Trichrome Stain Kit (ChemBio, CB6095.0200, İstanbul, Türkiye) was then applied. The sections were covered with Entellan® after final rinsing with the alcohol series and xylene.

#### Immunohistochemical staining

Bax (Santa Cruz Biotechnology catalog no: sc-23959, Heidelberg, Germany) and Bcl-2 (Santa Cruz Biotechnology catalog no: sc-7382, Heidelberg, Germany) antibodies were used for immunohistochemistry. Sections were deparaffinized in xylene for 30 min, incubated in Super Block (ScyTek Laboratories, Logan, UT) for 10 min, and washed with PBS. Primary antibody incubation was performed overnight, followed by PBS wash, secondary antibody incubation, and streptavidinperoxidase addition. The AEC (Sigma-Aldrich AEC101-1KT, Missouri, USA) chromogen was applied, and Mayer’s hematoxylin was used as a counterstain. Scoring of immune reaction intensity followed these criteria: no staining ‘0’, minimal ‘1’, moderate ‘2’, intense ‘3’ [6]. Scoring was based on cytoplasmic staining of Bax and Bcl-2 in 10 different seminiferous tubules from each preparation. All analyses were conducted by two blind investigators.

### RNA isolation and RT-qPCR analysis

RNA from testis samples was isolated using RiboEX reagent (Atlas Bio GeneAll, 301-001, Ankara, Türkiye). Samples were treated with DNase I (Thermo Fisher Scientific, #EN0521, Massachusetts, USA) to remove genomic DNA, followed by cDNA synthesis using the iScript™ cDNA Synthesis Kit (Gen Era A.S., Bio-Rad, 170-8891, İstanbul, Türkiye). Bax, Bcl-2, COL1A1, COL3A1 gene expression levels were quantified using 5× HOT FIREPol® EvaGreen® qPCR master mix (Solis BioDyne, Tartu, Estonia). Primer designs for the target and housekeeping (GAPDH) genes were generated using the IDT PrimerQuest tool. PCR was performed on a Bio-Rad CFX Connect™ Real-Time System following the protocol: 95 °C for 12 min, 95 °C for 15 s, 60 °C for 20 s, 72 °C for 20 s, over 40 cycles. The 2^(−ΔΔCT)^ method was used for quantitative analysis (Table 2).

### Biochemical analysis

#### Total antioxidant status (TAS)

TAS was measured with a fully automatic ABTS-based method (Rel Assay Diagnostics kit, Mega Tip, Gaziantep, Türkiye), expressed in μmol Trolox equivalent/l [18–20].

#### Total oxidant status (TOS)

TOS was measured with an automatic method based on the oxidation of the iron ion-o-dianisidine complex, expressed in μmol H_2_O_2_ equivalent/l (Rel Assay Diagnostics kit, Mega Tip, Gaziantep, Türkiye) [18–20].

#### Oxidative stress index (OSI)

The OSI was calculated as TOS/TAS, with TAS converted to μmol/l for normalization [18–20].

#### C-reactive protein (CRP)

CRP levels were measured by immunoturbidimetric analysis, with results in mg/l.

### Statistical analysis

Statistical analyses were performed using GraphPad Prism 8 (Boston, USA), with data expressed as mean ± SD. Data normality was tested with Shapiro-Wilk and One-Sample Kolmogorov-Smirnov tests. One-way ANOVA was used for parametric data, followed by Tukey’s HSD for pairwise comparisons. Non-parametric immunohistochemical data were analyzed using Kruskal-Wallis and Dunn’s tests. Significance was set at p<0.05.

## Results

### Histomorphological results

**Fig. 1:**
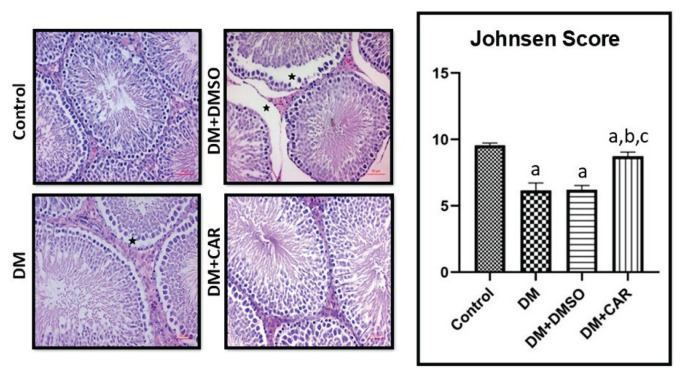
Structure of the testis in different groups of rats. In the control group, normal testicular architecture was observed. In the DM (Diabetes Mellitus) and DM+DMSO (Diabetes Mellitus+Di-methyl sulfoxide) groups, degeneration in seminiferous tubules and irregularities in germ cells (star) were noted. In the DM+CAR group, a normal seminiferous tubule structure was observed. Sections were stained with H&E. Scale bar = 50 μm. (**a**) Control, (**b**) DM, and (**c**) DM+ DMSO group, compared to which p<0.05 indicates statistical significance.

Sections of testicular tissue were stained with H&E and evaluated. Microscopic examination of the testicular sections in the control group revealed seminiferous tubules with normal structure, basal membrane with spermatogonia, scattered Sertoli cells, and a well-organized arrangement of germinal cells (spermatids, sperm cells) from the spermatogenic series. The interstitial connective tissue and Leydig cells in the interstitial area also appeared structurally normal. In contrast, the seminiferous tubules in the DM and DM+DMSO groups exhibited abnormal morphological features, including degeneration, atrophy, and disorganization of germ cells. However, in the DM+CAR group, the seminiferous tubules largely retained their original structure, and the overall histological appearance of the testis was nearly normal. Compared to the DM and DM+DMSO groups, some tubules in the DM+CAR group showed milder losses and disorganization in the germinal epithelial cells. Partial degenerative changes were observed in the Leydig cells in the interstitial areas (Fig. 1).

The average Johnsen scores for randomly selected 10 seminiferous tubules in all groups were evaluated based on the criteria provided in Table 1. According to this scoring, the DM, DM+DMSO, and DM+CAR groups showed statistically significant differences compared to the Control group (p<0.05). The DM+CAR group exhibited a significantly lower score compared to the Control group, while it was significantly higher compared to the DM and DM+DMSO groups (p<0.05) (Fig. 1).

### Masson’s trichrome staining of testicular tissue

**Fig. 2:**
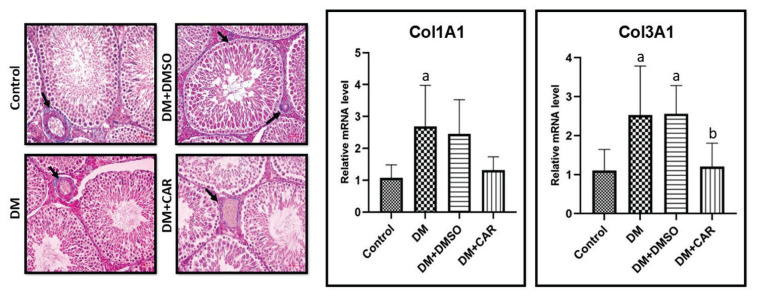
Testicular sections stained with Masson-Trichrome. Collagen fibers in the basal membrane and vascular walls are shown (arrow). Scale bar = 50 μm. (**a**) Control, (**b**) Compared to the DM+DMSO (Diabetes Mellitus+Dimethyl sulfoxide) group, p<0.05 indicates statistical significance.

Masson’s Trichrome staining was performed to visualize collagen fibers in connective tissue and fibrosis. In the control group, collagen fibers were observed in the vessel walls in the interstitial area. In the DM group, a slight increase in collagen fibers was noted in the vessel walls compared to the control. In the DM+DMSO group, an increase in collagen fibers was observed in the basal membranes in addition to the vessel walls. Lastly, in the DM+CAR group, a reduction in collagen fibers was observed in the vessel walls and basal membranes when compared to the DM and DM+DMSO groups (Fig. 2).

### mRNA expression analysis results of genes related to collagen synthesis

Analysis of COL1A1 mRNA expression showed a significant 2.49-fold increase in the DM group compared to the control (p=0.0241). In the DM+CAR-treated group, COL1A1 expression decreased 2.04-fold compared to the DM group (p=0.069) (Fig. 2).

For COL3A1 mRNA expression, a significant 2.27-fold increase was observed in the DM group compared to the control (p=0.0365). In the DM+CAR group, COL3A1 expression decreased 2.09-fold compared to the DM group (p=0.0555) (Fig. 2).

### Immunohistochemical results

**Fig. 3:**
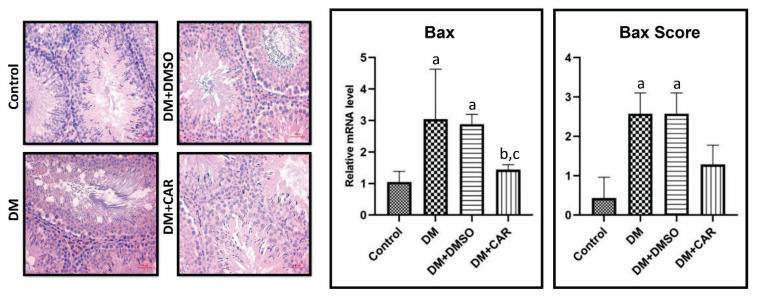
Bax immunohistochemical staining. Scale bar = 50 μm. Relative mRNA levels and scores: (**a**) Control, (**b**) DM (Diabetes Mellitus), and (**c**) Compared to the DM+DMSO (Diabetes Mellitus+Dimethyl sulfoxide) group, p<0.05 indicates statistical significance.

Immunohistochemical staining revealed weak Bax immunoreactivity in the control group. In the DM and DM+DMSO groups, a statistically significant increase in Bax immunoreactivity was observed. In the DM+CAR group, this increase was significantly reduced (Fig. 3).

### Results of RT-qPCR analysis

The effects of DM, DM+DMSO, and DM+CAR treatments on the mRNA expression of apoptosis-related genes (Bax, Bcl-2) in testicular tissue were analyzed using RT-qPCR. Results showed a 2.9-fold significant increase in Bax expression in the DM group compared to the control group (p=0.0026). The DM+CAR group had a 2.10-fold decrease in Bax expression compared to the DM group (p=0.0164) (Fig. 3).

**Fig. 4:**
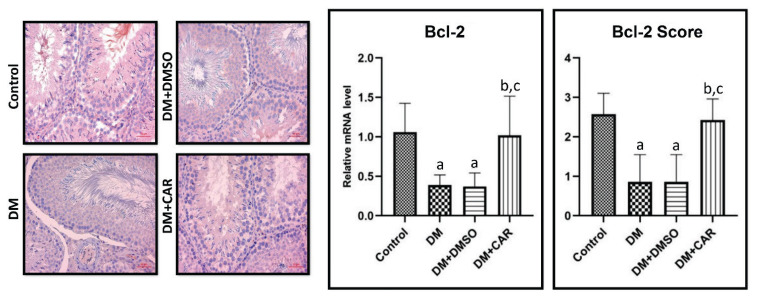
Bcl-2 immunohistochemical staining. Scale bar = 50 μm. Relative mRNA levels and scores: (**a**) Control, (**b**) DM (Diabetes Mellitus), and (**c**) Compared to the DM+DMSO (Diabetes Mellitus+Dimethyl sulfoxide) group, p<0.05 indicates statistical significance.

For Bcl-2 mRNA expression, analysis revealed a 2.72-fold significant decrease in the DM group compared to the control (p=0.0096). In the DM+CAR-treated group, a 2.6-fold significant increase in Bcl-2 mRNA expression was observed compared to the DM group (p=0.0152) (Fig. 4).

### Biochemical Analysis Results

**Fig. 5:**
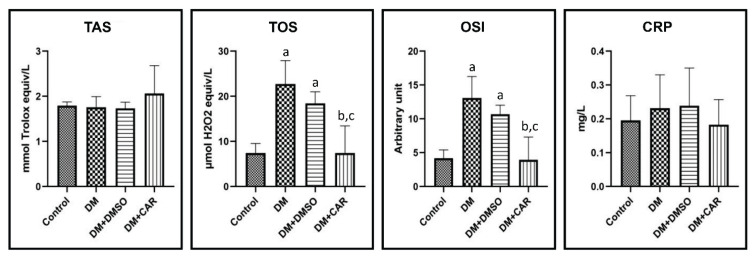
TAS (Total Antioxidant Status), TOS (Total Oxidant Status), OSI (Oxidative Stress Index) values in testicular tissue and CRP (C-Reactive Protein) measurement results in serum. (**a**) Control, (**b**) DM (Diabetes Mellitus), and (**c**) Compared to the DM+DMSO (Diabetes Mellitus+Dimethyl sulfoxide) group, p<0.05 indicates statistical significance.

TAS, TOS, OSI values in testicular tissue, and CRP levels in serum were measured. No statistically significant difference was found among the groups in terms of TAS values (p>0.05). For TOS values, a statistically significant increase was observed in the DM and DM+DMSO groups (p<0.05), while a reduction was noted in the DM+CAR group. In terms of OSI values, a statistically significant increase was detected in the DM and DM+DMSO groups, with a reduction in the DM+CAR group (p<0.05). No statistically significant difference was found in CRP levels among the groups (p>0.05) (Fig. 5).

## Discussion

In this study, where we examined structural changes, apoptotic markers, and oxidative parameters in the testicular tissue of male rats in the STZ-induced DM model, the therapeutic effects of CAR are noteworthy. Specifically, cellular losses in the testicular tissue, collagen accumulations, pro-apoptotic and anti-apoptotic markers, as well as oxidative findings, were improved or showed a tendency to improve in the CAR-treated group. Parallel to our findings, it is well known that DM has negative effects on testicular function and testosterone production at the cellular level, and that testicular dysfunction occurs due to oxidative stress in individuals with diabetes [21].

Shoorei *et al*. demonstrated in their diabetes model induced by STZ that increased oxidative stress led to testicular degeneration, which included damage to seminiferous tubules and loss of spermatogonia cells. In addition, they reported that the diameter of seminiferous tubules was significantly reduced, and their structure deteriorated, with a notable decrease in the average Johnsen score [2]. Zhao *et al*. showed in their study that Sertoli cells lost their supportive role because of diabetes, leading to germ cell loss [22]. Balci *et al*. demonstrated that CAR prevented morphological degeneration in seminiferous tubules caused by ischemia/reperfusion in testicular tissue [23]. Aksu *et al*. attributed the protective effect of CAR against cisplatin-induced reproductive damage to its antioxidant and anti-inflammatory properties [24]. Our findings support the results of these studies and demonstrate that DM leads to cellular losses, degeneration, and atrophy in the seminiferous tubules. However, CAR treatment showed a therapeutic effect against these impairments.

Studies on testicular interstitial fibrosis have shown that it disrupts the spermatogenic environment, impairing testosterone secretion and spermatogenesis, which can result in male infertility and sexual dysfunction [25,26]. Furthermore, some studies have identified that oxidative stress and inflammatory reactions caused by DM in testicular tissue can be inhibited by effective exogenous antioxidants and anti-inflammatory drugs [27,28]. El Helew *et al*. found a statistically significant increase in collagen fibers in the tunica albuginea, perivascular, and interstitial tissues of STZ-treated testis sections compared to the control group [29]. Previous studies have shown that CAR protects against liver damage and fibrosis induced by ethanol in rats [30]. Oxidative stress could be the cause of this fibrosis in testicular tissue, as ROS has been shown to convert fibroblasts into more synthetic myofibroblasts with excessive collagen production [31]. The results of our study indicated that CAR has a therapeutic effect on fibrosis increased by DM. However, while the COL1A1 level, determined through COL1A1 and COL3A1 mRNA levels, approached that of the control group with CAR treatment, it did not show a statistically significant difference when compared to the DM group. It is suggested that fibrosis induced by DM might be further suppressed with different CAR doses.

Oxidative stress is one of the causes of damage in spermatogenesis in DM, as hyperglycemia generates excessive oxygen radicals that cause significant damage to the sperm membrane, leading to lipid peroxidation and malondialdehyde (MDA) production [32]. In a study by Sun *et al*., free radicals were shown to accelerate germ cell death through apoptosis, reducing the number of germ cells [33]. Antioxidant therapy can regulate the delicate balance between pro-oxidant and antioxidant agents, thereby reducing oxidative damage. As a potent antioxidant molecule, CAR exhibits various biological and pharmacological properties and has been shown to reduce oxidative damage in the testicular tissue of diabetic animals [34]. Shoorei *et al*. demonstrated that CAR reduced ROS levels, decreased the intensity of Bax immunoreactivity, and increased antioxidant enzyme activity in the testicular tissue of diabetic rats [3]. Gur *et al*. showed that CAR had an anti-apoptotic effect by increasing Bcl-2 levels and reducing caspase-3 levels in testicular toxicity induced by sodium arsenite in rats [35]. Our study results showed that the expression of Bax, a pro-apoptotic marker that increases with DM, was downregulated by CAR treatment, suppressing it to the control group level. Conversely, Bcl-2, an anti-apoptotic marker that was downregulated in DM, was upregulated to control levels following CAR treatment. These findings highlight the crucial role of CAR, a potent antioxidant, in preventing apoptosis of testicular cells induced by oxidative damage in DM.

In another study by Daggulli *et al*., oxidative stress was induced in rat testes using a single dose of methotrexate (MTX). Following prophylactic CAR treatment, reductions in serum and testis MTX levels were observed. Additionally, after CAR treatment, lipid peroxidase activity and oxidative stress, which were previously elevated, significantly decreased. In the MTX+CAR group, lower levels of TOS, OSI, and MDA in serum and testis were observed compared to the MTX group. The MTX+CAR group also showed higher TAS levels in both serum and testicular tissue [36]. Numerous studies have shown that anti-inflammatory agents such as plants can suppress inflammation and oxidative stress, as well as improve reproductive function by increasing testosterone production [37]. Guvenc *et al*. demonstrated that different doses and long-term use of CAR and timol improved sperm quality and oxidative/antioxidative balance. In the same study, it was found that orally administered CAR reduced ROS levels in testicular tissue and increased antioxidant enzymes such as catalase (CAT) and glutathione peroxidase (GPx) [38]. In summary, it is well known that antioxidants help maintain the balance between oxidant and antioxidant molecules, providing protective and therapeutic effects against various pathophysiological conditions [39]. Our study results, in parallel with previous studies, demonstrated that DM induces oxidative stress, while CAR treatment alleviates this stress. This finding suggests that CAR supplementation may be beneficial for maintaining healthy testicular function in patients with DM.

CRP is a pentameric acute-phase reactant produced by the liver in response to proinflammatory cytokines, especially interleukin-6 [40,41]. Studies have documented increased CRP concentrations in individuals with diabetes compared to those without [42,43]. High CRP levels in diabetic individuals have been associated with a higher risk of mortality compared to those with normal CRP levels [44,45]. Akinboboye *et al*. found an association between elevated CRP levels and mortality in diabetic individuals, which was influenced by demographic characteristics, lifestyle behaviors, and medications [46]. Pérez-Segura *et al*. demonstrated that hsCRP levels were significantly higher in children diagnosed with DM1 compared to healthy controls [47]. Although studies have reported increased CRP levels in DM, our findings indicated that DM did not contribute to a statistically significant increase in CRP levels. The experimental animals experienced high blood glucose levels throughout the 4-week injection period. It is suggested that CRP levels may increase because of prolonged exposure to high blood glucose. Therefore, based on our study results, elevated CRP in DM may not be a parameter observed in the short term.

The findings of this study were obtained from experimental animals of the same age group. Therefore, the inability to assess the effects of age differences is a limitation of this study. Additionally, all animals with blood glucose levels above 270 mg/dl, the threshold for diabetes diagnosis, were included in the study, and blood glucose levels were not monitored afterward. Another limitation of our study is the lack of blood glucose monitoring during and after the injection period. Finally, another limitation of our study is the lack of testosterone level measurements within the scope of this study.

## Conclusions

In conclusion, the administration of CAR alleviated testicular dysfunction caused by diabetes, which is considered a leading cause of male infertility. CAR treatment was found to reduce the increased ROS levels, enhance antioxidant enzyme activity, and downregulate Bax while upregulating Bcl-2 at the gene and protein expression levels. Furthermore, CAR treatment also reduced fibrosis in the testicular tissue due to damage. These results highlight CAR’s potential to preserve fertility and improve testicular health, offering a promising foundation for future therapeutic approaches.

## Figures and Tables

**Table 1:** Johnsen Score for the evaluation of spermatogenesis in rats.

Score	Evaluation of spermatogenesis
*1*	No cells visualized in tubular cross section
*2*	Sertoli cells only
*3*	Only spermatogonia present
*4*	No sperm cells or spermatids, few spermatocytes (<5)
*5*	No sperm cells or spermatids, presence of spermatocytes
*6*	No sperm cells, few spermatids (<5 to 10)
*7*	No sperm cells, presence of spermatids
*8*	Presence of few sperm cells (<5 to 10)
*9*	Some sperm cells, with a disorganized epithelium
*10*	Compete spermatogenesis with mature sperm cells

**Table 2:** Primer sequences of apoptosis and collagen-related genes used in the RT-qPCR analysis.

*Gene*	Forward primer (5′→3′)	Reverse primer (5′→3′)
*BAX*	GATGGCCTCCTTTCCTACTTC	CTTCTTCCAGATGGTGAGTGAG
*BCL-2*	GGAGGATTGTGGCCTTCTTT	GTCATCCACAGAGCGATGTT
*COL1A*	CCAATGGTGCTCCTGGTATT	GTTCACCACTGTTGCCTTTG
*COL3A*	GTGTGATGATGAGCCACTAGAC	TGACAGGAGCAGGTGTAGAA
*GAPDH*	GCATTGCAGAGGATGGTAGAG	GCGGGAGAAGAAAGTCATGATTAG
